# Clinical Effects of Rehabilitation on Balance in People With Chronic Obstructive Pulmonary Disease: A Systematic Review and Meta-Analysis

**DOI:** 10.3389/fmed.2022.868316

**Published:** 2022-05-06

**Authors:** María Belén Canales-Díaz, Carolina Olivares-Valenzuela, Amanda Ramírez-Arriagada, Carlos Cruz-Montecinos, Jordi Vilaró, Rodrigo Torres-Castro, Rodrigo Núñez-Cortés

**Affiliations:** ^1^Department of Physical Therapy, Faculty of Medicine, University of Chile, Santiago, Chile; ^2^Section of Research, Innovation and Development in Kinesiology, Kinesiology Unit, San José Hospital, Santiago, Chile; ^3^International Physiotherapy Research Network (PhysioEvidence), Barcelona, Spain; ^4^Blanquerna School of Health Sciences, Global Research on Wellbeing, Universitat Ramon Llull, Barcelona, Spain; ^5^Department of Pulmonary Medicine, Hospital Clínic, University of Barcelona, Barcelona, Spain; ^6^Institut d'Investigacions Biomèdiques August Pi i Sunyer, Barcelona, Spain

**Keywords:** exercise, postural control, risk of fall, rehabilitation, chronic obstructive pulmonary disease (COPD)

## Abstract

**Background:**

Patients with chronic obstructive pulmonary disease (COPD) have systemic damage secondary to the primary pulmonary impairment, expressed in impaired peripheral musculature and a deficit in postural control compared to healthy subjects. This study aimed to determine the effects of rehabilitation on balance in patients with COPD.

**Methods:**

An exhaustive search was conducted in four databases (Pubmed, Cochrane Library, EMBASE, Web of Science). Articles with a population of COPD receiving rehabilitation (therapeutic exercise, pulmonary rehabilitation, or physical therapy modalities) in an outpatient setting were included. Two independent reviewers selected and assessed the study quality. The risk of bias was assessed with the Cochrane Risk of Bias Tool for Randomized Controlled Trials.

**Results:**

A total of eight studies involving 284 patients were included in the qualitative synthesis. The meta-analysis showed an overall result in favor of balance training for the Berg Balance Scale (mean difference 3.91 points; 95% CI: 1.51 to 6.31; *P* = 0.001), Timed Up and Go test (mean difference −1.58 s; 95% CI: −2.63 to −0.53; *P* = 0.003) and Unipedal stance test (mean difference 3.56 s, 95% CI: 2.58 to 4.54; *P*).

**Conclusion:**

This meta-analysis revealed that rehabilitation improve static and dynamic balance in patients with COPD.

**Systematic Review Registration:**

PROSPERO ID: CRD42020218367.

## Introduction

Non-communicable diseases kill more than 40 million people each year, accounting for 71% of deaths worldwide, of which respiratory diseases are the third most prevalent cause (1). Chronic Obstructive Pulmonary Disease (COPD) is the fourth leading cause of death in the world and the World Health Organization (WHO) estimates that it will be the third by the year 2030 (1). The COPD is defined as a common, preventable, and treatable disease characterized by respiratory symptoms and persistent airflow limitation due to airway or alveolar abnormalities, usually caused by significant exposure to noxious particles or gases (2). There is sufficient evidence to state that COPD patients have systemic damage secondary to the primary pulmonary impairment, which is expressed in an impairment of peripheral musculature and a deficit of postural control compared to healthy subjects of the same age (3).

Musculoskeletal dysfunction in COPD is associated with different factors, including nutritional alterations, inflammation, oxidative stress, drugs, and the presence of different comorbidities (4). On the other hand, physical deconditioning caused by exertional dyspnea leads to a more sedentary lifestyle, generating greater respiratory and peripheral muscle mass loss (5). In this context, muscle weakness, physical inactivity, and limited mobility are associated with more significant deterioration of postural control in people with COPD, which is associated with increased mortality, less independence, a poorer quality of life, and a higher risk of falling (3, 6). Regarding the latter, it has been reported that the history of falls in people with COPD could range from 33 to 50% (7–10). In routine practice, different clinical tests can be used to predict the fall risk in patients with COPD. For example, the Timed Up and Go test has high reliability and predictive validity for falls in older people (11), and the Berg Balance Scale (BBS) has been identified as useful in successfully identifying individuals at risk for falls (12).

The traditional approach to treating this disease has been based on alleviating and/or improving respiratory symptomatology (13). Nevertheless, in recent decades, pulmonary rehabilitation protocols have been modified to provide more comprehensive and functional care to patients, focusing on increasing participation, minimizing health care costs, increasing exercise tolerance, improving quality of life, decreasing hospitalizations, and reducing mortality (14). In addition, balance training has been installed as a new treatment target in COPD patients to prevent falls (15).

A recent meta-analysis found that people with COPD have reduced balance compared to healthy subjects, which may be related to reduced muscle strength, physical activity, and exercise capacity (16). Although pulmonary rehabilitation has shown promising results in improving exercise capacity (17), outcomes related to balance have been little studied. Therefore, this study aimed to determine the effects of rehabilitation on static and functional balance in people with COPD.

## Methods

This systematic review was conducted following the Preferred Reporting Items for Systematic Reviews and Meta-Analyses (PRISMA) recommendations (18). The protocol was previously registered in the PROSPERO International Prospective Register of Systematic Reviews (CRD42020218367) in November 2020.

### Eligibility Criteria

Inclusion criteria were based on PICO: P) Population: Adults with a confirmed diagnosis of COPD based on Global Obstructive Lung Disease (GOLD) criteria (2); I) Intervention: outpatient rehabilitation programs (e.g., therapeutic exercise, pulmonary rehabilitation, or physical therapy interventions); C (Comparison): conventional treatment, usual care or active controls (e.g., education) or no intervention; O (Outcome): The included studies should evaluate static or dynamic balance using clinical tests [i.e., Berg Balance Scale (BBS), Time up and go (TUG), Unipedal Stance Test (UST), Balance Evaluation Systems Test (BESTest), or similar]. Randomized controlled trials (RCTs), controlled intervention studies, and before and after (Pre-Post) studies were included. Patients with neurological conditions or patients with acute exacerbation of COPD in the last 4 weeks were excluded.

### Search Strategy

An exhaustive search was conducted in the following databases: PubMed, Cochrane Library, EMBASE and Web of Science, with the keywords divided into four domains: (1) Population: chronic obstructive pulmonary disease OR COPD; (2) Intervention: rehabilitative interventions OR pulmonary rehabilitation OR treatment outcome OR physical therapy modalities OR physical therapy interventions; (3) Outcomes: postural balance OR accidental falls OR risk of falls. (4) Condition: adults OR elderly. No temporary or language filters were included. The search was performed on titles, abstracts, and keywords. The selected terms will be combined using Boolean logical operators (OR, AND, NOT). All references were analyzed using Rayyan web software (19). An additional hand search of the references included in the selected studies and in the previous systematic reviews was performed.

### Selection of Studies

First, two independent reviewers (COV and ARA) screened the studies by title and abstract according to the eligibility criteria. A third reviewer (RNC.) resolved discrepancies, and references considered not relevant were discarded. After this selection, full-text articles were accessed to assess compliance with the eligibility criteria. Any discrepancies were resolved by consensus in consultation with a third reviewer (RNC). The exclusion criteria were: (1) Wrong study design: Letters to the editor, editorial, review articles, and *in vivo* and *in vitro* studies were excluded (including the type of wrong publication); (2) Wrong population: we excluded non-outpatients or patients with non-stable diseases; (3) Wrong outcome: Measurement of balance by instrumental tests (e.g., posturography).

### Data Extraction

A Microsoft Excel (Microsoft^®^ Excel 2010, Microsoft Corporation, Seattle, USA) table was designed for data extraction. Data extraction was performed in duplicate using a standardized form that included the following data: author, year of publication, country of origin, study design, number of patients, number of men and women, age, forced expiratory volume in the first second in percent predicted values (FEV_1_%pred), intervention (frequency, follow-up), results and conclusions. Disagreements were resolved by a third reviewer (RNC). If any relevant data were not included in the article, the authors were contacted by e-mail to obtain the information.

### Methodological Quality Assessment

Risk of bias assessment of the included studies was performed using the Cochrane Risk for bias (RoB) tool for randomized clinical trials and ROBINS-I for non-randomized clinical trials (20). Three reviewers (COV, ARA, MCD) performed the assessment independently, and if there were discrepancies or disagreements between the reviewers' judgments, a fourth reviewer (RNC) was consulted.

### Data Synthesis and Analysis

RevMan 5.3 software (The Cochrane Collaboration, Oxford, UK) was used for meta-analysis and generation of a forest plot that showed combined estimates with a 95% confidence interval. The mean difference of the results and the standard deviation were pooled for each study comparing an experimental intervention with a control group. Then, a random effects model with inverse variance (IV) method was used to obtain the combined effect measures for each primary outcome. This choice of weight minimizes the imprecision (uncertainty) of the pooled effect estimate. Statistical heterogeneity was assessed by I^2^ and classified as could be unimportant (I^2^ = 0-40%), moderate (I^2^ = 30-60%), substantial (I^2^ = 50-90%) or considerable (I^2^ = 75-100%) (20).

## Results

### Study Selection

**Figure 1:**
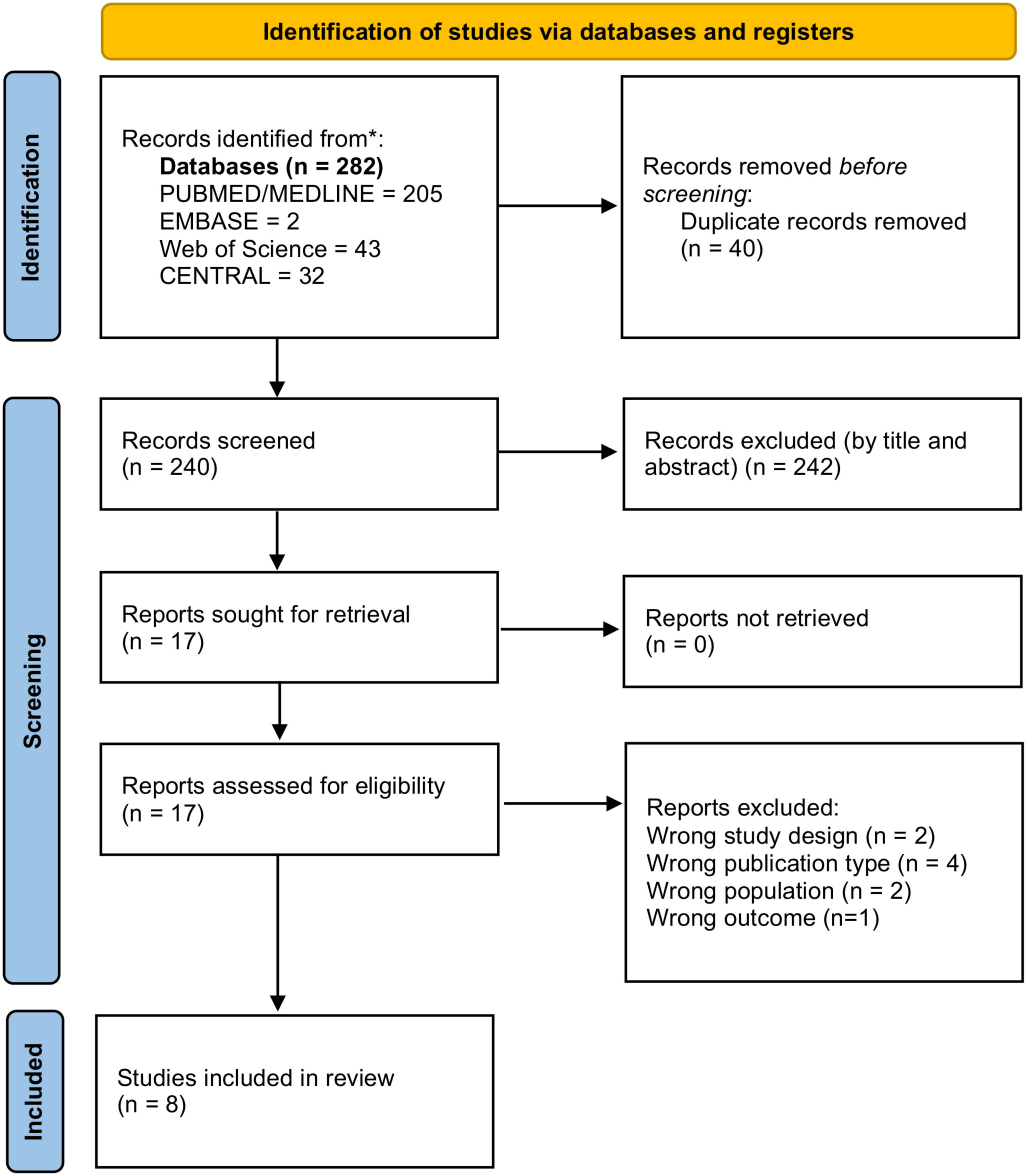
Study selection process.

The initial search yielded 282 articles of interest, then 40 duplicates were eliminated and 225 were excluded in the screening of titles and abstracts. Of the 17 articles evaluated in the full-text screening, four were eliminated due to type of publication, two due to design, two due to study population, and one due to outcome. Finally, a total of eight articles were included in the qualitative synthesis (21–28). A detailed diagram of the selection process of these articles is presented in Figure 1.

### Characteristics of the Included Studies

The included articles were published between 2014 and 2020, of which six corresponded to randomized clinical trials (21–25, 28) one non-randomized clinical trials (26), and one quasi-experimental study (27). Of the total number of articles selected, three were from Europe, two from Africa, two from Asia and one from South America (Table 1). All were published in English.

**Table 1:** Characteristics of the studies.

**References**	**Country**	**Design**	***n* (M/F)**	**Age (years)**	**FEV_**1**_ (% Pred)**	**BMI (kg/m^**2**^)**	**Follow-up**
Jácome et al. (27)	Portugal	Before-After	Total: 26 (16/10)	Total: 67.8 ± 10.3	Total: 83.8 ± 6.4	Total: 28.7 ± 5.0	12 weeks
Marques et al. (26)	Portugal	Before-After	Total: 22 (13/9)	Total: 68.0 ± 11.8	Total: 72.2 ± 22.3	Total: 28.4 ± 6.0	12 weeks
Mkacher et al. (23)	Tunisia	RCT	Total: 68 (68/0) CG: 33 (33/0) IG: 35 (35/0)	IG: 58.2 ± 4.3 CG: 61.2 ± 3.2	IG: 39.4 ± 10.3 CG: 38.6 ± 8.6	IG: 24.1 ± 3.8 CG: 25.2 ± 2.6	24 weeks
Rinaldo et al. (22)	Italy	RCT	Total: 24 (24/0) IG: 12 (12/0) CG: 12 (12/0)	IG: 66.2 ± 4.2 CG: 66.1 ± 4.5	IG: 60.1 ± 24.3 CG: 72.2 ± 18.8	IG: 29.9 ± 4.4 CG: 28.4 ± 5.7	42 weeks
Mekki et al. (21)	Tunisia	RCT	Total: 45 (45/0) IG:25 (25/0) CG: 20 (20/0)	IG: 59.6 ± 4.8 CG: 59.5 ± 3.1	IG: 57.7 ± 14.4 CG: 57.1 ± 10.2	IG: 25.6 ± 0.7 CG: 25.6 ± 0.5	24 weeks
Mounir et al. (28)	Egypt	RCT	Total: 48 (48/0) IG:24 (24/0) CG:24 (24/0)	IG: 63.1 ± 1.7 CG: 62.4 ± 1.6	IG: 63.6 ± 5.6 CG: 61.6 ± 8.5	IG: 24.8 ± 2.2 CG: 24.9 ± 2.4	8 weeks
Suresh et al. (25)	United Arab Emirates	RCT	Total: 20 (15/5) IG: 10 (8/2) CG: 10 (7/3)	IG: 55.2 ± 3.4 CG: 55.2 ±- 4.6	NR	NR	16 weeks
de Castro et al. (24)	Brazil	RCT	Total: 31 (18/13) CG: 17 (9/8) IG: 14 (9/5)	CG: 64 ± 8 IG: 65 ± 8	CG: 48 ± 17% IG: 51 ± 15%	IG: 28 ± 5 CG: 27 ± 4	12 weeks

*BMI, Body mass index; CG, Control group; F, Female; IG, Intervention group; FEV_1_%pred, Forced expiratory volume in the first second in percent predicted values; M, Male; NR, Not reported; RCT, Randomized controlled trial*.

### Participants

In total, 284 patients with COPD were enrolled in the included studies. The sample size was between 20 (25) and 68 (23) participants, with mean age varied between 55.2 ± 3.4 and 68.0 ± 11.8 years. Four studies included only male subjects with COPD (21–23, 28). Overall, most of the included patients were male (*n* = 247, 87%). The BMI ranged from 24.1 ± 3.8 to 29.9 ± 4.4 and FEV1%pred ranged from 38.6 ± 8.6% to 83.8 ± 6.4%. Total follow-up time ranged from 8 to 24 weeks.

### Summary of Results

Seven of the selected studies included combined treatment protocols (21–26, 28), while only one study had an isolated intervention protocol (27). Regarding the intervention performed, can be classified into the following categories: balance exercises (22, 23, 25, 27, 28), strength training (21–28), endurance or walking exercises (21, 22, 24–28), aquatic exercises (24), and exercises with neuromuscular electrical stimulation (21). In addition, three articles included education and psychosocial support sessions (22, 23, 27). Table 2 summarizes the different therapeutic interventions (frequency, intensity, time and type, follow-up), results, and conclusions used in each of the included studies.

**Table 2:** Synthesis of interventions and results.

**References**	***n***	**Intervention program**	**Frequency**	**Results**	**Conclusion**
Jácome et al. (27)	26	PR program with exercise training: Endurance training (walking) at 60–80% of the average speed achieved during the 6MWT (20 min); Strength training including seven exercises (2 sets of 10 repetitions) of the major upper and lower limb muscle groups using free weights and ankle weights (15 min); Psychoeducation (90 min w/session); Balance training (5 min); Psychoeducation (one session/week, 90 min).	PR: 3 sessions/week, 60 min each	Significant effects on TUG: 7.8 vs. 6.7 seconds (*P* < 0.001, ES 0.8).	The PR program was effective in improving dyspnea, functional balance, muscle strength, exercise tolerance and cardiovascular endurance in patients with mild COPD.
Marques et al. (26)	22	Endurance, Strength and Balance exercise training + psychosocial support and education (60 min each session): Warm-up (5-10 min); Endurance: walking at 60-80% of HR obtained in 6MWT (20 min); Strength: 7 exercises of 2 sets of 10 repetitions for upper and lower extremities with 50–85% of 10 RM (15 min); Balance: static and dynamic exercises, using postures that gradually reduce the base, dynamic movements that disturb the center of gravity, tension of postural muscle groups, and dynamic movements with secondary tares decreasing the base of support (5 min); Return to calm.	Exercise: 3 times a week for 12 weeks	Significant post-PR improvements in TUG score (mean change −1.7 ± 1.4 s; *P* = 0.001; effect size = 1.249).	PR with a specific balance training component had a large effect on functional balance in COPD patients.
Mkacher et al. (23)	68 CG: 33 IG: 35	Balance: duration of 30 min. Four types of exercise: posture exercise, transitions, walking exercises and functional strength. PR: twice daily supervised exercise training, daily breathing exercises, self-management education, psychological and social support.	3 days a week for 24 weeks.	Significant differences between groups were observed in TUG (*P* < 0.01), Tinetti (*P* < 0.01), BBS (*P* < 0.01) and Unipedal Stance Test scores. (*P* < 0.05).	Balance training incorporated into PR has significant improvements in balance test scores in COPD patients.
Rinaldo et al. (22)	24 IG: 12 CG: 12	IG: Physical activity education program with a progressive increase in the pace of physical activity in three modalities: aerobic classes with flexibility and balance exercises, Nordic walking or non-weight bearing exercises in circuit training. CG: Structured exercise program (traditional). Self-monitored intensity. The protocol included aerobic and strength exercises for 60 min. Endurance: 30 min of cycling or treadmill at modified Borg intensity 3-4; Strength: 4 sets of legs, arms and trunk at 50-80% of 1RM, load was adjusted cad 3 or 4 weeks according to results. Each session ends with flexibility and balance exercises.	IG: 60 min session, 3 times per week, for 28 weeks. CG: Prescribed program for 14 weeks.	Balance control improved markedly in both groups after training but was not maintained at follow-up.	Both programs can effectively and safely improve health-related parameters in COPD patients.
Mekki et al. (21)	45 IG:25 CG:20	IG: neuromuscular electrical stimulation + PR. CG: PR only PR: Warm-up (5-10 min), joint movement, stretching and low-intensity exercise, breathing techniques; Endurance: 45 min on cycle ergometer. 60-70% effort of max. HR obtained in 6MWT; Strength: 15 min. four exercises of 2 sets of 10 repetitions for upper and lower limbs; Return to calm. 45 min. Neuromuscular electrical stimulation (20 min) for quadriceps femoris, triceps suralis and bilateral hamstring. Applied current: biphasic symmetrical rectangular pulses of 400 μs with a frequency of 50 Hz. With intensity ranging from 15 to 60 mA.	3 times a week for 24 weeks.	In IG, TUG and BBS values are significantly higher than CG (*P* = 0.02, *P* = 0.01, respectively); Improved mid-lateral center of pressure displacement in I (*P* < 0.001).	Neuromuscular electrical stimulation added to PR improves physical tolerance and balance compared to PR alone.
Mounir et al. (28)	48 IG: 24 CG: 24	IG: balance training + PR CG: PR only Balance: Functional strength exercise (e.g., heel raise, toe raise, walking on toes, step-ups in all directions, squats, and core strength on ball); Stance exercise (e.g., tandem, narrow, one leg stance, and stand on uneven surfaces) with open eyes (each exercise 30 s) and then with eyes closed (each exercise 15 s); Transition exercise; Gait training. PR: Endurance training based on 60–80% of 6MWT speed achieved; Strength exercise using Thera band and weights (3 sets 8 repetitions each), 50–75% of 1 RM, repeated at week 4; daily breathing exercises.	IG: 25-30 minutes (total session), three times a week (every other day). CG: 25-30 minutes (total session), three times per week (day after day).	Significant increase in the BBS and BESTest after treatment in both groups, with a percentage of improvement in the control group was 5.01 and 9.15%, respectively, whereas in the study group was 16.04 and 25.46%, respectively.	Addition of balance training to PR program was more effective in improving balance in elderly patients with COPD.
Suresh et al. (25)	20 IG: 10 CG: 10	IG: Balance Training + PR. CG: PR only PR: 60 min. Endurance: Borg 5-6 for dyspnea or fatigue, walking; Strength: biceps, triceps, deltoids, quadriceps, hip flexors, extensors and abductors, 10-15 reps and Borg. Balance training: 15-20 min. circuit (standing exercises, transitions, ambulatory and functional exercises for balance).	3 days a week for 8 weeks.	Significant differences (*p* < 0.05) between groups before and after intervention for BBS and TUG	PR with or without balance training in subjects with moderate COPD produces statistically and clinically significant effects on balance, exercise tolerance, health-related quality of life, and risk of falls.
de Castro et al. (24)	31 CG: 17 IG: 14	IG: Aquatic training in the pool at 33° (water level: 1 m). CG: Land training. Endurance training: cycling and walking with sound stimuli. Cycling according to perceived exertion (Borg 4-6); Strength training: quadriceps, biceps and triceps (70% of 1RM, fully submerged segment).	3 days a week (60 min. session) for 12 weeks.	Aquatic training positively affected functional balance (TUG: mean difference of −1.17 s, 95%CI: −1.93 to −0.41, *P* = 0.006). In contrast, the static balance remained unchanged in both groups.	Functional balance improved after three months of high-intensity exercise training performed in water. However, non-specific training independent of the environment appears insufficient to improve static balance.

*6MWT, 6-min walking test; BBS, Berg Balance Scale; BESTest, The Balance Evaluation Systems Test; COPD, chronic obstructive pulmonary disease; CG, control group; ES, Effect size; HR, Heart frequency; IG, intervention group; PR, Pulmonary rehabilitation; RM, repetition maximum; SGRQ, St George Respiratory Questionnaire; TUG, Timed up and Go*.

### Berg Balance Scale

**Figure 2:**
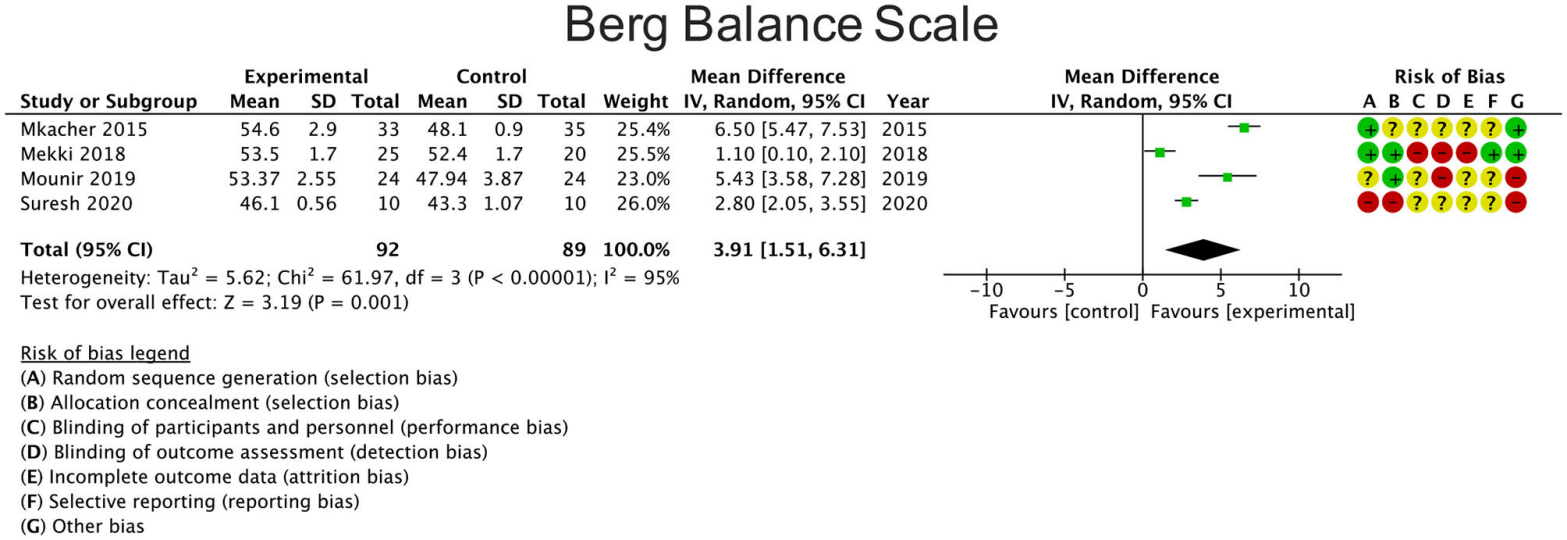
Effects of rehabilitation interventions on the Berg balance scale. Each study considered in the meta-analysis corresponds to a point estimate, which is bounded by a 95% CI.

Four studies reported changes in balance using the BBS comparing the results with a control group (21, 23, 25, 28). The overall result of the meta-analysis was in favor of the experimental group [mean difference 3.91 points (95% CI: 1.51 to 6.31; *P* = 0.001)] (Figure 2). Heterogeneity between studies was considerable (I^2^ = 95%).

### Timed Up and Go

**Figure 3:**
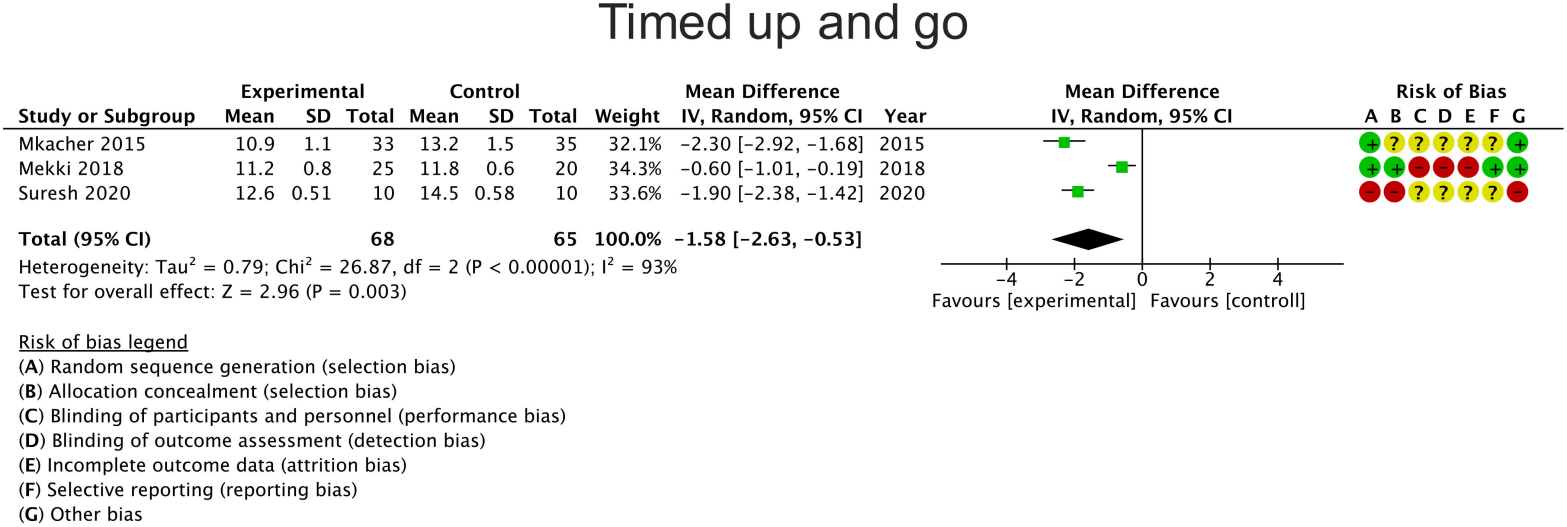
Effects of rehabilitation interventions on the Test timed up and go. Each study considered in the meta-analysis corresponds to a point estimate, which is bounded by a 95% CI.

Six studies reported changes in balance using the TUG, of which three studies were included in the meta-analysis (21, 23, 25) as they compared the intervention with a control group. The overall result of the meta-analysis was in favor of the experimental group [mean difference −1.58 s (95% CI: −2.63 to −0.53; *P* = 0.003)] (Figure 3). Heterogeneity between studies was considerable (I^2^ = 93%).

### Other Balance Measures

**Figure 4:**
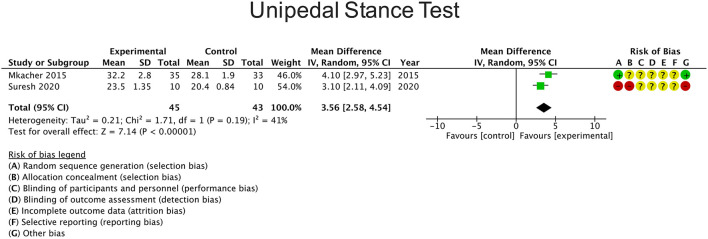
Effects of rehabilitation interventions on the Unipedal stance test. Each study considered in the meta-analysis corresponds to a point estimate, which is bounded by a 95% CI.

Four studies reported changes in balance using the UST of which two studies were included in the meta-analysis (23, 25) as they compared the intervention with a control group. The overall result of the meta-analysis was in favor of the experimental group [mean difference 3.56 s (95% CI: 2.58 to 4.54; *P*)] (Figure 4). Heterogeneity between studies was moderate (I^2^ = 41%). On the other hand, two studies used the Activities-Specific Balance Confidence (ABC) scale (23, 25), one study used the Balance Evaluation Systems Test (BESTest) (28) and one study used the Tinetti test score (23). These studies reported a significant improvement in balance compared to the control group.

### Risk of Bias

**Figure 5:**
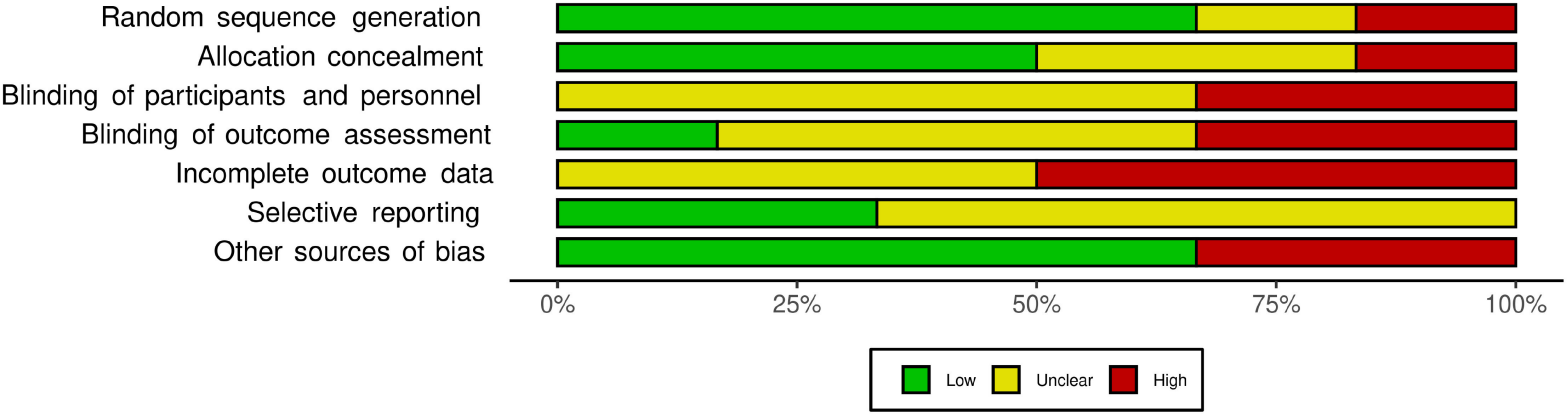
Summary of the risk of bias assessment using the Cochrane Risk for bias (RoB) tool.

Regarding the random sequence generation domain, only one study was found to be at high risk (25). In the allocation concealment domain, one study presented high risk (25) and two were uncertain (23, 24). Two studies presented high risk for blinding of participants and personnel (21, 24) while the rest of the studies were uncertain (22, 23, 25, 28). Two studies presented high risk for blinding of outcome assessment domain (21, 28) and three studies had a high risk of bias due to incomplete outcome data (21, 22, 24). None of the studies presented a high risk of bias in the selective reporting domain, but most of the judgment was uncertain (22, 23, 25, 28). Two studies presented high risk for other sources of bias (25, 28). Figure 5 shows the summary of each Risk of Bias domain.

## Discussion

This systematic review collected information regarding physical therapy interventions and protocols focused on balance training and postural control in COPD patients. Most of the studies that added balance training found significant changes for balance improvement in COPD patients compared to conventional treatments.

Different interventions have been proposed in the current literature to improve balance, including strength training (21–28) endurance or walking exercises (21, 22, 24–28), aquatic exercises (24), exercises with neuromuscular electrical stimulation (21), and specific balance training in addition to traditional treatments (22, 23, 25, 26, 28). Significant improvements obtained in balance were evaluated with different clinical tests for functional balance (TUG) (21, 23, 25), static balance (UST) (22–25), or both (BBS and BESTestest) (21, 23, 25).

The minimum clinically important difference (MCID) with respect to TUG was recently established between 0.9 and 1.4 s (29). Therefore, our results indicate that balance training had an overall significant and clinically relevant effect for this outcome (mean difference −1.58 s). Regarding BBS, although the overall effect obtained from the meta-analysis (mean difference 3.91 points) was larger than the minimum detectable change described in the literature (3.49 points) (30), anchor-based MCID estimates range from 3.5 to 7.1 for BBS (31), making it difficult to estimate whether this change was clinically relevant. Regarding UST, the overall effect obtained from the meta-analysis (mean difference 3.56 s) was lower than the minimal detectable change (4.03 s) established in patients with COPD (30). The unipedal stance training protocols are effective for promoting balance gains in healthy adults (32). However, in COPD patients future studies are needed to establish the effectiveness regarding volume, frequency, and potential progressions of unipedal stance exercise protocols' (32).

These results agree with two similar systematic reviews; Delbressine et al. (33) found that exercise-based interventions have the potential to improve balance in COPD patients and pulmonary rehabilitation combined with balance training showed greater benefits. Chuatrakoon et al. (34) also found that available RCTs suggest that exercise interventions (e.g., cycling, Tai Chi) can improve balance performance in COPD patients, both in the outpatient and inpatient settings. However, in contrast to both reviews (33, 34), our study performed a quantitative synthesis of the effects of rehabilitation interventions on balance. This allows objective data to be obtained on the clinical relevance and accuracy of the differences between the proposed rehabilitation interventions and their comparators. In addition, our meta-analysis only included balance assessment by clinical testing in the outpatient setting, excluding studies that incorporated instrumental assessment or evaluations in the inpatient setting. Therefore, our results could be extrapolated to a primary health care context, where community-based rehabilitation appears as a possibility for constant treatment that allows permanent control, and at the same time, promotes the autonomy of individuals concerning their pathology (35).

The various clinical tests used indicate that an alteration in static or functional balance can increase the risk of falls in patients with COPD. In particular, this population presents an even higher risk of falls due to musculoskeletal disorders and age. Furthermore, the pharmacological treatment of COPD includes corticosteroids (2), and it has been reported that this group of drugs favors the production of osteoclasts and a decrease in osteocytes, leading to an increased risk of osteopenia or osteoporosis (36, 37). Therefore, the management of these risk factors must be comprehensive.

Given that falls are associated with an increased risk of all-cause mortality in patients with COPD (38), improving balance and preventing falls should become a priority treatment goal in these patients. In this context, we know that balance can be influenced by many factors, including muscle strength and cognitive aspects (39). In this context, new approaches to assess and improve postural control have been proposed in the literature, such as dual-task training (40–42). This proposed intervention (e.g., secondary tasks, counting backwards) was considered in only two of the included studies (23, 26), with a large effect on functional balance in COPD patients. Therefore, pulmonary rehabilitation programs should include all these aspects in future research.

This study has some limitations. In general, the sample size included in the studies was relatively small. In addition, most of the studies had a high risk of bias in some of the domains evaluated, the most critical being the incomplete reporting of results, blinding of the patient and staff, and blinding of the evaluator. Finally, despite the significant improvements obtained in meta-analyses for the overall effect size in each outcome, we observed substantial to considerable statistical heterogeneity, which could be attributed to the few studies included and the low sample size.

## Conclusion

Our results revealed that rehabilitation improve static and dynamic balance in patients with COPD. Due to the small sample size in this meta-analysis and considering the high prevalence of falls in people with COPD, future large-scale randomized controlled trials are needed to evaluate different exercise protocols' efficacy to prevent falls.

## Data Availability Statement

The original contributions presented in the study are included in the article/Supplementary Material, further inquiries can be directed to the corresponding author/s.

## Author Contributions

CC-M and RN-C: conception and design. MC-D, CO-V, AR-A, RT-C, and RN-C: acquisition, analysis, and interpretation of data. MC-D, CO-V, AR-A, and RN-C: manuscript writing. CC-M, JV, and RT-C: critical revision of the manuscript. All authors read and approved the final manuscript.

## Conflict of Interest

The authors declare that the research was conducted in the absence of any commercial or financial relationships that could be construed as a potential conflict of interest.

## Publisher's Note

All claims expressed in this article are solely those of the authors and do not necessarily represent those of their affiliated organizations, or those of the publisher, the editors and the reviewers. Any product that may be evaluated in this article, or claim that may be made by its manufacturer, is not guaranteed or endorsed by the publisher.
