# Supplementary material for: Clinical Effects of Rehabilitation on Balance in People With Chronic Obstructive Pulmonary Disease: A Systematic Review and Meta-Analysis
Source: Front Med (Lausanne). 2022 May 6;9:868316. doi: 10.3389/fmed.2022.868316 (PMC9120665; doi:10.3389/fmed.2022.868316)
Supplement: Supplementary file 1 [file Table_1.docx]

**PubMed/MEDLINE**

Date of search: December 18, 2020.

| # | **Search strategy** | **Results** |
| --- | --- | --- |
| #1 | ((pulmonary disease)[Title/Abstract] OR (chronic obstructive)[Title/Abstract] OR (COPD))[Title/Abstract] | 1,117,602 |
| #2 | ((rehabilitative interventions)[Title/Abstract] OR (pulmonary rehabilitation)[Title/Abstract] OR (treatment outcome)[Title/Abstract] OR (physical therapy modalities)[Title/Abstract] OR (physical therapy interventions))[Title/Abstract] | 1,762,958 |
| #3 | ((postural balance)[Title/Abstract] OR (accidental falls)[Title/Abstract] OR (risk of falls))[Title/Abstract] | 62,846 |
| #4 | ((adults)[Title/Abstract] OR (elderly))[Title/Abstract] | 8,236,114 |
| #5 | #1 + #2 + #3 + #4 + #5  ((pulmonary disease)[Title/Abstract] OR (chronic obstructive)[Title/Abstract] OR (COPD)) [Title/Abstract] AND ((rehabilitative interventions)[Title/Abstract] OR (pulmonary rehabilitation)[Title/Abstract] OR (treatment outcome)[Title/Abstract] OR (physical therapy modalities)[Title/Abstract] OR (physical therapy interventions)) [Title/Abstract] AND ((postural balance)[Title/Abstract] OR (accidental falls)[Title/Abstract] OR (risk of falls)) [Title/Abstract] AND ((adults)[Title/Abstract] OR (elderly))[Title/Abstract] | 205 |

**Cochrane Central Register of Controlled Trials**

Date of search: December 18, 2020.

| # | **Search strategy** | **Results** |
| --- | --- | --- |
| #1 | ((pulmonary disease) OR (chronic obstructive) OR (COPD)) in Title Abstract Keyword | 35,734 |
| #2 | ((rehabilitative interventions) OR (pulmonary rehabilitation) OR (treatment outcome) OR (physical therapy modalities) OR (physical therapy interventions)) in Title Abstract Keyword | 391,475 |
| #3 | ((postural balance) OR (accidental falls) OR (risk of falls)) in Title Abstract Keyword | 10,128 |
| #4 | ((adults) OR (elderly)) in Title Abstract Keyword | 682,177 |
| #5 | #1 + #2 + #3 + #4 + #5  ((pulmonary disease) OR (chronic obstructive) OR (COPD)) AND ((rehabilitative interventions) OR (pulmonary rehabilitation) OR (treatment outcome) OR (physical therapy modalities) OR (physical therapy interventions)) AND ((postural balance) OR (accidental falls) OR (risk of falls)) AND ((adults) OR (elderly)) in Title Abstract Keyword | 32 |

**EMBASE**

Date of search: December 18, 2020.

| # | **Search strategy** | **Results** |
| --- | --- | --- |
| #1 | 'pulmonary disease':ab,ti OR 'chronic obstructive':ab,ti OR copd:ab,ti | 139,961 |
| #2 | 'rehabilitative interventions':ab,ti OR 'pulmonary rehabilitation':ab,ti OR 'treatment outcome':ab,ti OR 'physical therapy modalities':ab,ti OR 'physical therapy interventions':ab,ti | 44,768 |
| #3 | 'postural balance':ab,ti OR 'accidental falls':ab,ti OR 'risk of falls':ab,ti | 6,503 |
| #4 | adults:ab,ti OR elderly:ab,ti | 1,103,837 |
| #5 | #1 + #2 + #3 + #4 + #5  ('pulmonary disease':ab,ti OR 'chronic obstructive':ab,ti OR copd:ab,ti) AND ('rehabilitative interventions':ab,ti OR 'pulmonary rehabilitation':ab,ti OR 'treatment outcome':ab,ti OR 'physical therapy modalities':ab,ti OR 'physical therapy interventions':ab,ti) AND ('postural balance':ab,ti OR 'accidental falls':ab,ti OR 'risk of falls':ab,ti) AND (adults:ab,ti OR elderly:ab,ti) | 2 |

**WEB OF SCIENCE**

Date of search: December 18, 2020.

| # | **Search strategy** | **Results** |
| --- | --- | --- |
| #1 | (((pulmonary disease) OR (chronic obstructive) OR (COPD))) | 243,890 |
| #2 | (((rehabilitative interventions) OR (pulmonary rehabilitation) OR (treatment outcome) OR (physical therapy modalities) OR (physical therapy interventions))) | 699,599 |
| #3 | (((postural balance) OR (accidental falls) OR (risk of falls))) | 61,802 |
| #4 | (((adults) OR (elderly))) | 1,786,244 |
| #5 | #1 + #2 + #3 + #4 + #5  (((pulmonary disease) OR (chronic obstructive) OR (COPD)) AND ((rehabilitative interventions) OR (pulmonary rehabilitation) OR (treatment outcome) OR (physical therapy modalities) OR (physical therapy interventions)) AND ((postural balance) OR (accidental falls) OR (risk of falls)) AND ((adults) OR (elderly))) | 43 |
